# Differential diagnosis of chorea (guidelines of the German Neurological Society)

**DOI:** 10.1186/s42466-023-00292-2

**Published:** 2023-11-23

**Authors:** Carsten Saft, Jean-Marc Burgunder, Matthias Dose, Hans Heinrich Jung, Regina Katzenschlager, Josef Priller, Huu Phuc Nguyen, Kathrin Reetz, Ralf Reilmann, Klaus Seppi, Georg Bernhard Landwehrmeyer

**Affiliations:** 1 Department of Neurology, St. Josef-Hospital, Huntington-Zentrum NRW, Ruhr-Universität Bochum, Bochum, Germany; 2 Department of Neurology, Schweizerisches Huntington-Zentrum, Bern University, Bern, Switzerland; 3 Kbo-Isar-Amper-Klinikum Taufkirchen/München-Ost, Munich, Germany; 4 Department of Neurology, University Hospital Zürich, Zurich, Switzerland; 5 Department of Neurology, Karl Landsteiner Institute for Neuroimmunological and Neurodegenerative Disorders, Klinik Donaustadt, Vienna, Austria; 6 Department of Psychiatry and Psychotherapy, Klinikum Rechts der Isar, School of Medicine and Health, Technical University of Munich, Munich, Germany; 7 Neuropsychiatry, Charité-Universitätsmedizin Berlin, Berlin, Germany; 8 Department of Human Genetics, Huntington-Zentrum NRW, Ruhr-Universität Bochum, Bochum, Germany; 9 Department of Neurology, Euregional Huntington Centre Aachen, RWTH Aachen University Hospital, Aachen, Germany; 10 George-Huntington-Institute, Muenster, Germany; 11 Department of Radiology, Universitaetsklinikum Muenster (UKM), Westfaelische Wilhelms-University, Muenster, Germany; 12 Department of Neurodegenerative Diseases and Hertie-Institute for Clinical Brain Research, University of Tuebingen, Tuebingen, Germany; 13 Department of Neurology, Medical University of Innsbruck, Innsbruck, Austria; 14 Department of Neurology, Huntington’s Disease Centre, Ulm University, Ulm, Germany

## Abstract

**Introduction:**

Choreiform movement disorders are characterized by involuntary, rapid, irregular, and unpredictable movements of the limbs, face, neck, and trunk. These movements often initially go unnoticed by the affected individuals and may blend together with seemingly intended, random motions. Choreiform movements can occur both at rest and during voluntary movements. They typically increase in intensity with stress and physical activity and essentially cease during deep sleep stages. In particularly in advanced stages of Huntington disease (HD), choreiform hyperkinesia occurs alongside with dystonic postures of the limbs or trunk before they typically decrease in intensity.

**Summary or definition of the topic:**

The differential diagnosis of HD can be complex. Here, the authors aim to provide guidance for the diagnostic process. This guidance was prepared for the German Neurological Society (DGN) for German-speaking countries.

**Recommendations:**

Hereditary (inherited) and non-hereditary (non-inherited) forms of chorea can be distinguished. Therefore, the family history is crucial. However, even in conditions with autosomal-dominant transmission such as HD, unremarkable family histories do not necessarily rule out a hereditary form (e.g., in cases of early deceased or unknown parents, uncertainties in familial relationships, as well as in offspring of parents with CAG repeats in the expandable range (27–35 CAG repeats) which may display expansions into the pathogenic range).

**Conclusions:**

The differential diagnosis of chorea can be challenging. This guidance prepared for the German Neurological Society (DGN) reflects the state of the art as of 2023.

## Introduction

This guideline is an abridged and translated short version of the guidance on chorea and Huntington’s Disease prepared for the German Neurological Society, covering diagnostic aspects as well as symptomatic treatment options. A complete version of this guideline (in German) can be found on the website of the Deutsche Gesellschaft für Neurologie (www.dgn.org/leitlinien) and the AWMF (Arbeitsgemeinschaft wissenschaftlicher Medizinischer Gesellschaften).

AWMF registry No. Is 030/028; level of guideline: S2k; last update: November 24th, 2022; Valid until: November 23th, 2027; source: https://register.awmf.org/de/leitlinien/detail/030-028

This guideline has been approved by the German Neurological Society (DGN) and the German Association for Psychiatry, Psychotherapy, and Psychosomatics; German Society of Human Genetics; Swiss Neurological Society; Austrian Society of Neurology, and was reviewed by the German HD Association (Deutsche Huntington-Hilfe e.V.)

### What´s new?

The differential diagnosis of chorea can be complex. Taking a family history can be helpful in many cases. A variety of new mutations causing chorea as a symptom has been described. For sporadic cases, it can be helpful to differentiate based on: course, age at disease onset, symmetry, additional neurological, clinical and paraclinical findings such as concurrent ataxia, polyneuropathy, cutaneous or scleral telangiectasias, liver pathology, laboratory abnormalities or specific MRI findings.

### Guidelines in detail

The recommendations of this guideline were established through a Delphi process (strength of consensus > 75–95% for all recommendations), achieved over two rounds of voting. All statements for which consensus strength is not specified were met with a consensus of > 95%.

#### Hereditary disease entities presenting with chorea or featuring choreiform movements

- Huntington’s disease [14, 95]
- C9orf72 expansion mutation (frontotemporal dementia, motor neuron disease and movement disorders, probably frequent phenocopy of HD [43])
- Spinocerebellar ataxia 17 (corresponds to Huntington‘s-disease-like-4; HDL4 [95])
- Spinocerebellar ataxia types 3, 2, 1 and 7 [78]
- Spinocerebellar ataxia type 8 [95]
- Spinocerebellar ataxia type 12 (mainly India [48, 95])
- Spinocerebellar ataxia type 48/SCAR 16 due to mutations in the STUB1 gene [61, 91]
- Dentato-rubro-pallido-Luysian atrophy (mainly Japan, DRPLA; [78])
- Ataxia telangiectasia and ataxia telangiectasia like disease (serum alpha-fetoprotein ↑ [14])
- Ataxia with oculomotor apraxia (AOA1 (serum albumin ↓) and AOA2 (serum alpha-fetoprotein↑; now also called SCAN2 [2, 14, 57])
- McLeod syndrome (CK↑, ± acanthocytes in blood smear↑, myocardial abnormalities, striatal atrophy, analysis of the Kell/Kx blood group phenotype or mutations in XK gene [25])
- Chorea-acanthocytosis (CK↑, ± acanthocytes in blood smear↑, striatal atrophy, ChoreinWestern blot (LMU, Munich), detection of mutation in the CHAC gene (VPS13A [25, 95])
- Huntington’s disease-like 2 (HDL2; predominantly in patients of African origin [14, 95])
- Benign hereditary chorea (including thyroid transcription factor 1 gene, TITF1/NKX2-1; L-Dopa or methylphenidate therapy might be helpful [32, 106])
- ADCY5 mutation [13, 14]

#### Other rare inherited disease entities

- Huntington’s disease-like 1 and 3, only described in individual families [14]
- HDL1 with prion protein (PrP) gene mutations (PRNP) and rapid progression [14, 95]
- HDL3, a family [47]
- RNF216 mutation (autosomal recessive, leukoencephalopathic lesions and possibly Serum gonadotropin ↓ [93])
- ANO3 mutations [52]
- FRRS1L mutations (Saudi Arabia; also epilepsy [95])
- Primary Familial Brain Calcification (formerly “Fahr’s disease”, cMRI/CCT helpful (SLC20A2-,PDGFB, PDGFRB or XPR1 gene [95])
- POLG gene mutations (dystonia, myoclonus, discrete chorea [101])
- Leigh's disease [63]
- SETX mutation (with motor neuron disease [94])
- Laurence-Moon-Biedl-Bardet syndrome [65]
- Friedreich ataxia [41]
- NBIA “neurodegeneration with brain iron accumulation” (umbrella term for e.g. Pantothenate kinase-associated neurodegeneration (PKAN 2), neuroferritinopathies (FTL), Aceruloplasminemia (CP), phospholipase-associated neurodegeneration (PLAN), Betapropeller protein-associated neurodegeneration (WDR45), infantile neuroaxonal dystrophy (PLA2G6), C19orf12, C2orf37, FA2H, ATP13A2, COASY and DCAF17 mutations—more likely no chorea)) with iron deposits in the basal ganglia as a typical MRI finding [3, 14, 82, 95, 103, 113])
- Wilson's disease [14, 95]
- TAR DNA binding protein variation (TARDBP; with frontotemporal dementia [51])
- Lesch-Nyhan syndrome; X-linked [1, 14]
- Niemann-Pick type C [46]
- Cereoid lipofuscinosis [72]
- Lipidoses, aminoacidosis and carbohydrate metabolism disorders [69, 96]
- Phenylketonuria [44]
- Paroxysmal kinesiogenic dyskinesia (PKD; paroxysmal kinesiogenic choreoathetosis; dystonia 10 [14, 50], weekly parenteral doses of vitamins and minerals might be helpful [10])
- Paroxysmal non-kinesiogenic dyskinesia (PNKD); dystonia type 8 [14, 50]
- Paroxysmal choreoathetosis with infantile febrile convulsions; ICCA [14]
- Tuberous brain sclerosis [116]
- FUS-related ALS [34]
- Mutations in iron-responsive element-binding protein 2; IREB2 [20]
- 18p deletions syndrome [22]
- X-linked Dystonia-Parkinsonism; Lubag; Dyt3 [31]
- FXTAS [56]
- Dopamine D2 receptor variant (also dystonia [107])
- GLRB mutations (GlyR β-subunit; with hyperekplexia [29])
- CAMK4 variant (with dystonia, autism, developmental delay, later chorea [120])
- Eukaryotic translation elongation factor (EEF1A2) mutations (also associated with epilepsy, autism, intellectual impairment, sudden onset of chorea [55])
- ERCC4 gene mutations in xeroderma pigmentosa (with ataxia [15])
- Replication factor complex subunit 1 (RFC1) mutations (cerebellar ataxia, neuropathy, vestibular areflexia syndrome (CANVAS) and leading cause of late-onset ataxia, 11% Chorea [105])
- Polynucleotide kinase phosphatase (PNKP) mutation (rather benign course, early onset, with microcephaly, epilepsy, developmental delay, ataxia with oculomotor apraxia (AOA type 4) and polyneuropathy [12])
- “Benign chorea type 2” (with onset of the disease around the age of 40; Japan [97])

#### Hereditary chorea primarily presenting in children and adolescents

- NKX2-1, ADCY-5, FOXG1, GNAO1, GPR88, SLC2A1, SQSTM1, ATP8A2 or SYT-1 Mutations [5]
- Hereditary disorders of glycosylation (CDG; in children [71])
- ELAC2 gene mutations (rare mitochondrial disease with cardiomyopathy, children with developmental delay, possibly acanthocytes [76])
- SCN2A mutation (neonatal, early childhood epilepsy, developmental disorders, possibly autism and episodic ataxia [115])
- PDE10A mutations (MRI with bilateral striatal lesions [68])
- KCNQ2 mutations (associated with fever [27])
- ATP1A3 mutations (alternating hemiplegia of childhood (AHC), rapid-onset dystonia, parkinsonism, CAPOS (cerebellar ataxia, areflexia, pes cavus, optic atrophy, sensorineural hearing loss [100])
- ATP1A2 mutations (regression, hemiplegia, epilepsy [11])
- SUCLA2 mutation in mitochondrial DNA (hypotonia, Dyston/Leigh-like syndrome, deafness, but also myopathy, ataxia [36])
- Glutaric aciduria (AR [44])
- ARX loss (intellectual impairment [87])

#### Autoimmune and paraneoplastic choreiform syndromes

Sydenham's chorea (chorea minor, post-streptococcal infection disease): anti-streptolysin O (ASL), anti-DNAse B (anti-streptodornase B, ASNB). The relevance of anti-basal ganglia antibodies (immunohistochemical analysis [38] and dopamine D2 receptor Ab (cell-based testing is unclear; so far laboratory analysis established in one centre; specificity not clearly proven [24].

Systemic lupus erythematosus (SLE); antiphospholipid antibody syndrome (APS); Chorea gravidarum (often SLE, APS or other autoimmune diseases are discussed as the underlying cause for chorea gravidarum [14, 86]).

Multiple sclerosis [37, 104]

Behçet's disease [40].

Chorea in synaptic (idiopathic and paraneoplastic) autoimmune encephalitis is possible, but rarely isolated. GAD65-AK; CASPR2-Ab; NMDA-R-AK; CRMP-5 IgG [108], N-type or P/Q-type calcium channel antibodies [17, 70]; Anti-SOX1-Ab [117], LGI-1-Ab (30–60 high-frequency, short daily brachiofacial dystonic seizures per day preceding or paralleling limbic encephalitis), and probably also other autoimmune, partly post-infectious or -vaccine encephalitis, e.g. B. IgLON5-Ab (usually with sleep disorders and stridor); [39, 60, 74, 88, 110].

Very rare: paraneoplastic chorea with antibodies against onconeural (intracellular) antigens, most of these cases are progressive and multisystemic syndromes with subacute onset (anti-CV2/CRMP-5, Anti-Hu, Anti-Yo, Anti-Ma2 [14, 21, 30].

Phosphodiesterase 10A antibody [6]; Takayasu vasculitis [62]; Rasmussen syndrome [35]; celiac disease with anti-gliadin antibodies [112]; steroid responsive Encephalopathy in Autoimmune Thyroiditis (SREAT [102]); Microscopic polyangiitis (MPA [45]).

#### Infectious causes

HIV encephalopathy [84]; viral encephalitis (mumps, measles, varicella-zoster virus, herpes simplex virus, ECHO group viruses); new variant of Creutzfeldt-Jakob disease; diphtheria; bacterial endocarditis; neurobrucellosis; neurosyphilis; neuroborreliosis; other bacterial encephalitides; cerebral toxoplasmosis; CNS cryptococcosis; neurocysticercosis [14]; Whipple's disease (including ataxia, vertical gaze palsy, oculomasticatory myorhythmias, cognitive impairment [79]); subacute sclerosing panencephalitis (SSPE [98]); Influenza A encephalopathy [81]; SARS-CoV-2 encephalitis [42].

#### Structural lesions of the basal ganglia

Ischemic or hemorrhagic infarcts; neoplasms; abscessing lesions (incl. toxoplasmosis abscesses and tuberculomas); demyelinating lesions; central pontine/extrapontine myelinolysis; neurosarcoidosis [4, 14]; cavernoma [83]; structural lesions, may also cause hemichorea. Vascular encephalopathy, lacunar infarcts may also cause intermittent chorea [92].

#### Metabolic, endocrine and toxic causes

Nonketotic hyperglycemia in diabetes mellitus (T1-weighted MRI sequences often show localized hyperintensity, especially in the putamen [18]).

Hypoglycemia; hypo/hypernatremia; hypocalcemia; hypoparathyroidism (sometimes presenting as hemichorea and with calcification of the basal ganglia [26]; hyperthyroidism; acute intermittent porphyria; liver failure including chronic hepatocerebral degeneration; kidney failure; carbon monoxide; manganese; mercury; thallium; organophosphates, 3-NP [14]; vitamin B12 deficiency [28]; Wernicke encephalopathy [89]; 3-Hydroxy-sobutyryl-CoAHydrolase (HIBCH) deficiency (children, sometimes only paroxysmal chorea, e.g. during stress [99]), Tay-Sachs disease [59].

#### Medication and drug-induced chorea

Dopamine receptor antagonists (e.g. phenothiazine, butyrophenone, benzamide) including antiemetics (metoclopramide); medications for the treatment of Parkinson's disease (such as L-dopa, dopamine agonists, anticholinergics); antiepileptic drugs (e.g. phenytoin, carbamazepine, valproic acid, gabapentin, lamotrigine, pregabalin [85], levetiracetam [118]; calcium channel blockers (cinnarizine, flunarizine, verapamil); lithium; tricyclic antidepressants; SSRI [54]; anti-malarial drugs; steroids; oral contraceptives; antihistamines (H1 and H2); psychostimulants (methylphenidate, amphetamines, pemoline, cocaine); baclofen, digoxin, cyclosporine, theophylline [14]; buphrenorphine, hydromorphone and other opiates [66]; ceftriaxone [119]; memantine [9].

#### Other causes

Polycythaemia vera [67]; essential thrombocythaemia [109]; post-pump chorea after cardiac surgery [90]; superficial siderosis [58]; Moyamoya disease [16]; chorea gravidarum (idiopathic or secondary,see under: “Autoimmune and paraneoplastic choreiform syndromes” [49]; Covid-19 vaccination [7]; intoxications with wood or plant protection products, e.g. propiconazole [77].

### Important differential diagnoses for the syndrome chorea include

1. Focal epilepsy [33]. Chorea can sometimes be mistaken for focal epilepsy.
2. Tic Disorders: Unlike chorea, tic disorders are characterized by typical premonitory urges and the ability to suppress tics for a short time [8].
3. Akathisia: It can be challenging to differentiate akathisia from chorea, especially since many antidopaminergic drugs including presynaptic dopamine depletors, such as tetrabenazine, can induce akathisia. If akathisia is suspected, a reduction in antihyperkinetic medication may be indicated. Propranolol, anticholinergics, benzodiazepines, and postsynaptic serotonin-5-HT2a receptor antagonists like ritanserin, cyproheptadine, trazodone, mianserin, or mirtazapine can be helpful in managing drug-induced akathisia [80].
4. Myoclonus-dystonia disorders: Conditions like epsilon-sarcoglycan (SGCE) DYT11 gene mutations (often alcohol-sensitive) or VPS16 gene mutations can present as myoclonus-dystonia disorders [75]. Additionally, many other conditions discussed above may exhibit a myoclonus-dystonia phenotype (e.g., benign chorea or PKAN2).

### Suggested work-up in a patient with chorea

Please refer to Table 1. Medical history, particularly family history, medication history, presence of other relevant medical conditions (as mentioned above).

**Table 1:** Overview of key recommendations for the differential diagnosis of chorea (modified from: Cardoso et al. [14], Hermann and Walker [44], Nguyen et al. [73], Schneider and Bird [95]

Pattern of inheritance	Autosomal-dominant	Huntington's disease (most common inherited chorea, generally with positive Family history and typical clinic, molecular genetic testing can be carried out as a next step; but ~ 8% without positive family history [111] C9orf72 mutations Spinocerebellar ataxia type 3, 2, 1, 7, 8, 12, 17, 48 DRPLA (especially Japan) HDL2 (especially of African origin) Neuroferritinopathy (NBIA) NKX2-1 (benign course of the disease)
	Autosomal-recessive	Wilson’s disease Neuroacanthocytosis Syndroms, VPS13A- and XK-disease /McLeod (CK, blood smear, chorein western blot) PLAN, PKAN2, aceruloplasminemia (NBIA) Friedreich’s Ataxia Niemann-Pick type C disease AOA1, AOA2 (now SCAN2), AT (AFP elevantion, Albumin reduced) Bilateral striatal necrosis, glutaric aciduria and similiar diseases in childhood
	X-linked	McLeod-Syndrome (CK, blood smear, Kx and Kell blood group phenotype) FXTAS Lesch-Nyhan-Syndrome RETT Syndrome Metabolic diseases in childhood
According to course	Acute	Stroke/ICB
	Subacute	Metabolic Paraneoplastic Drug side effects Malignancies Prion diseases
	Chronic progressive	Neurodegenerative
	Not progressive	Drug side effects Benign Chorea (NKX2-1)
	Episodic	Paroxysmal dyskinesias (PED, SLC2A1*,* Dyt 18)
Presenting predominantly in childhood (a selection)		Benign hereditary chorea (including thyroid-transcription-factor-1-gene, TITF1/NKX2-1 mutations; L-Dopa or methylphenidate treatment potentially helpful [32, 106] ADCY5 mutation [13, 14] Paroxysmal dyskinesias (PED, SLC2A1, Dyt 18) NBIA Lesch-Nyhan-Syndrome, X-linked [44] RETT-Syndrome, X-linked [44] Mitochondriopathies [44] Polynucleotide kinase phosphatase (PNKP) mutation (rather benign course, early onset, with microcephaly, epilepsy, developmental delay, ataxia with oculomotor apraxia (AOA type 4) and polyneuropathy [12] ELAC2 gene mutations, rare mitochondrial disease with cardiomyopathy, children with developmental delay, possibly acanthocytes [76] FOXG1-, GNAO1-, GPR88-, SLC2A1-, SQSTM1-, ATP8A2-oder SYT-1-mutation [5] Hereditary disorders of glycosylation (CDG; in children [71] SCN2A mutation (neonatal, early childhood epilepsy, developmental disorders, possibly autism and episodic ataxia [115] PDE10A mutations, MRI with bilateral striatal lesions [68] KCNQ2 mutations, associated with fever [27] ATP1A3 mutations, alternating hemiplegia of childhood (AHC), rapid-onset dystonia, parkinsonism, CAPOS (cerebellar ataxia, areflexia, pes cavus, optic atrophy, sensorineural hearing loss [100] ATP1A2 mutations with regression, hemiplegia, epilepsy [11] SUCLA2 mutations, mitochondrial DNA, hypotonia, Dyston/Leigh-like syndrome, deafness, but also myopathy, ataxia [36] Glutaric aciduria; AR [44] Non-inherited: Sydenham chorea
Symmetry	Asymmetric in diseases with structural lesion or metabolic cause (also generalized possible)	Stroke/ICB Post-pump chorea (after heart surgery) Infantile cerebral palsy Vasculitis, Moyamoya, multiple sclerosis, autoimmune diseases Tumor/structural lesion Polycythaemia vera Non-ketotic hyperglycaemia Chorea minor, antiphospholipid antibodies, drug-induced [19]
Signs on	Asymmetric	structural lesion
	Subcortical dementia/Frontal lobe syndrome	Neurodegenerative
	Ataxia	SCA 1–3, 7, 8, 12, 17, 48, DRPLA, AOA Typ I/II and others
	Loss of reflexes/CK	Neuroacanthocytosis syndroms
	Seizures	Juvenile Huntington’s disease, neuroacanthocytosis Syndromes (VPS13A- and XK-disease/McLeod), kinesigenic dyskinesia
MRI findings	Iron deposits	NBIA (chorea especially: PKAN 2, neuroferritinopathies (FTL), aceruloplasminemia)
	Calcium depositis (formerly “M. Fahr”)	Primary Familial Brain Calcification (SLC20A2-,PDGFB, PDGFRB others), parathyroid hormone disorders, possibly mitochondriopathy
	Leukenzephalopathy	RNF216
	Atrophy pattern	Caudate atrophy in HD, cerebellar atrophy in ataxia
	Structural lesion	Ischemic or hemorrhagic infarcts; neoplasms
Recommen-ded laboratory tests	Especially in sporadic cases	Routine lab, including liver parameters, CK (neuroacanthocytosis, but also after a fall, possibly blood smear asking for acanthocytes), vitamin B12, methyl-maleonate, ferritin, AFP (increased in AT and AOA II), albumin (decreased in AOA I) antistreptolysin (AST), Anti-DNAse B, Ceruloplasmin, Copper in serum and 24-h urine collection, ANA, ENA, antidouble-strand DNA (dsDNA), ANCA, RF, anti-gliadin Ab, paraneoplastic or antineuronal antibodies: e.g.: Anti-HU, -Yo, -Ma, -CRMP-5/CV2, anti-NMDA-Rec-Ab, anti-GAD-, anti-Iglon5-, anti-LGl-1-, phospholipid-Ab, cardiolipin-Ab, TSH (basal), anti-thyroid peroxidase (MAK) Ab, TSH receptor auto Ab (TRAK), parathyroid hormone, erythropoietin and hematocrit (Polycythemia vera), Treponema pallidum screening test, borrelia IgG/IgM Ab, HIV, possibly pregnancy test

Neurological assessment to determine if chorea is the only presenting movement disorder and if additional neurological or systemic signs are present. In general, chorea is often more noticeable during conversations, especially when emotionally charged, such as during discussions of stressful topics or during neuropsychological testing. However, during the actual neurological examination, chorea may be less pronounced [8].

Cerebral imaging studies (MRI, if contraindicated CT scan, asking for focal lesions or caudate and/or cortical atrophy. Note that for the determination of caudate atrophy, coronal sections should be available. Signal changes in T2-weighted imaging? Contrast-enhancing lesions? Hypointensities suggesting iron deposition on iron-sensitive magnetic resonance images in order to rule out symptomatic causes or provide evidence for pathognomonic alterations (e.g. "Eye of the tiger" sign for PKAN2; hypointensities in the basal ganglia, particularly in bilateral globus pallidus and substantia nigra for NBIAs; "Face of the giant panda" sign for Wilson's disease; cerebellar or pontine atrophy for hereditary ataxia).

Depending on the findings and circumstances: Comprehensive laboratory testing, including cerebrospinal fluid analysis to explore the differential diagnoses mentioned above. FDG-PET-CT or FDG-PET-MRI for tumor screening in suspected paraneoplastic etiology. Heavy Metal Assessment (mercury, manganese, thallium), "drug test" in serum and/or urine.

Additional tests in selected cases: Positron Emission Tomography (e.g., FDG-PET) to detect reduced glucose utilization/hypometabolism in basal ganglia (e.g., in HD and other neurodegenerative disorders causing chorea) or increased glucose utilization/hypermetabolism in Sydenham's chorea or autoimmune encephalitis including SLE with cerebral involvement, associated with hypometabolism in the prefrontal and premotor cortex [53, 114], hypermetabolism in the hippocampus and orbitofrontal cortex [64], and potentially parieto-occipital hypometabolism in patients with neuropsychiatric disorders [23], contralateral striatal hypoperfusion in patients with non-ketotic hyperglycemia.

### If Huntington’s disease is suspect

Molecular genetic testing (determination of CAG repeats in the Huntingtin gene, Chromosome 4p) after informed consent following the Genetic Diagnosis Act (GenDG).

Unified Huntington’s Disease Rating Scale (UHDRS’99) total motor score.

Neuropsychological or Behavioral Neurological Assessment (psychomotor slowing, frontal-executive dysfunction, memory impairment, decreased speech fluency, spatial-visual disturbances, formal cognitive testing according to UHDRS).

Psychiatric Examination (personality changes, changes in motivation, irritability, aggression, depression, suicidal ideation, delusions, hallucinations, obsessive–compulsive disorders, and sexual disorders; it is recommended to use the "Problem Behaviour Assessment" scale (PBA-s), which is also used in the ENROLL-HD observational study).

## Data Availability

Not applicable.
